# Review on Bee Products as Potential Protective and Therapeutic Agents in Male Reproductive Impairment

**DOI:** 10.3390/molecules26113421

**Published:** 2021-06-05

**Authors:** Joseph Bagi Suleiman, Ainul Bahiyah Abu Bakar, Mahaneem Mohamed

**Affiliations:** 1 Department of Physiology, School of Medical Sciences, Universiti Sains Malaysia, Kubang Kerian 16150, Kelantan, Malaysia; bagisuleiman@yahoo.com (J.B.S.); ainul@usm.my (A.B.A.B.); 2 Department of Science Laboratory Technology, Akanu Ibiam Federal Polytechnic, Unwana P.M.B. 1007, Afikpo, Ebonyi State, Nigeria; 3 Unit of Integrative Medicine, School of Medical Sciences, Universiti Sains Malaysia, Kubang Kerian 16150, Kelantan, Malaysia

**Keywords:** bee products, preventive, therapeutic, male reproductive impairment

## Abstract

Bee products are sources of functional food that have been used in complementary medicine to treat a variety of acute and chronic illnesses in many parts of the world. The products vary from location to location as well as country to country. Therefore, the aim of this review was to identify various bee products with potential preventive and therapeutic values used in the treatment of male reproductive impairment. We undertook a vigorous search for bee products with preventive and therapeutic values for the male reproductive system. These products included honey, royal jelly, bee pollen, bee brood, apilarnil, bee bread, bee wax, and bee venom. We also explained the mechanisms involved in testicular steroidogenesis, reactive oxygen species, oxidative stress, inflammation, and apoptosis, which may cumulatively lead to male reproductive impairment. The effects of bee pollen, bee venom, honey, propolis, royal jelly, and bee bread on male reproductive parameters were examined. Conclusively, these bee products showed positive effects on the steroidogenic, spermatogenic, oxidative stress, inflammatory, and apoptotic parameters, thereby making them a promising possible preventive and therapeutic treatment of male sub/infertility.

## 1. Introduction

Honeybees produce various products containing many biochemical components such as minerals, vitamins, and polyphenols, which are biologically active [1]. These compounds have served as preventive and therapeutic agents in the last four decades and have been used in apitherapy [2]. Bee products are used for the treatment of some conditions such as multiple sclerosis, arthritis, wounds, pain, gout, shingles, burns, tendonitis, and infections [3]. Therefore, apitherapy being a simple, convenient, and available method is practiced in traditional self-heath care and also holds promise for the treatment of periodontal diseases, mouth ulcers, and other diseases of the oral cavity as well [4]. The bee products include bee venom, honey, pollen, royal jelly, propolis, bee bread, bee brood, and beeswax, which are produced by four types of insects: honeybees (Apis), stingless bees, honey wasps, and honey ants [5]. Usually, honey bees are of four species, namely *A. mellifera*, *A. cerana*, *A. dorsata*, and *A. florea*.

Honey is a light or dark amber liquid formed by bees from the nectar of flowers [6], while propolis is a sticky, greenish–brown product used as a coating to build their hives. The royal jelly is a milky substance that contains water, proteins, sugar, fats, vitamins, salts, and amino acids. Similarly, bee pollen is a pellet from flower pollen gathered by worker honeybees and used as the nutritional sources for the beehive. Additionally, bee venom is an acidic colorless liquid made up of enzymes, sugars, minerals, and amino acid, beeswax is a mixture of pollen oils and wax to form a yellow or brown color, while bee bread is a mixture of pollen and nectar or honey [7,8,9,10,11].

Meanwhile, nowadays, there are many studies investigating the potential protective and therapeutic roles of these bee products in health, including male infertility [12,13,14,15]. The World Health Organization guidelines revealed that 15–25% of couples struggle to conceive, and approximately half of these cases are caused by infertility in males due to alteration in sperm concentration, motility, and/or morphology, which is present in samples collected [16]. Several mechanisms have also been identified as possible cause(s) of infertility, which include defects in the steroidogenic pathway, the imbalance in the pro and antioxidant activity, the irregularities in the apoptotic pathway, the imbalance of the pro and anti-inflammatory markers, and the generation of the reactive oxygen species. The articles used for this review were searched in the following databases: PubMed, Science Direct, Springerlink, EBSCOHOST, SCOPUS, and Google Scholar, from 2000 to 2021. The keywords (bee products, preventive, therapeutic, male reproductive impairment) in single or in combination were also searched in in these databases (as listed above). A total of 1150 articles were identified, and 114 articles met the inclusion criteria of having the key words. To the best of our knowledge, no article has looked at the various studies on the effects of bee products on the male reproductive impairment; hence, the aim of this review was to look at the effects of various bee products as potential protective and therapeutic agents in male reproductive impairment.

## 2. Factors Involved in Male Reproductive Impairment

The male reproductive tract goes through impairment as a result of testicular steroidogenic dysfunction, apoptosis, oxidative stress, and inflammation, and it is occasioned by metabolic diseases (diabetes and obesity), heavy metals (cadmium, lead, and mercury) as well as stress (heat and exercises).

### 2.1. Testicular Steroidogenesis Dysfunction

The endocrine system is responsible for the production of steroid hormones in various tissues such as the testis, ovaries, and adrenal glands, and the process is referred to as steroidogenesis. However, of these steroid hormones, testosterone, luteinizing hormone (LH), and follicle-stimulating hormone (FSH) are the main regulators of spermatogenesis [17,18,19]. Meanwhile, the major androgen found in the male testis is testosterone, which regulates the reproductive organ functions in males. Testicular steroidogenesis has been well documented in both animal [20,21] and clinical [22,23,24] studies in which a decrease in the activity level of androgenic enzymes in the testis leads to the decreased level of testosterone in the serum and testis, respectively. This is usually observed in various metabolic diseases and other chronic stressful activities. Conversely, decreases in levels of FSH and LH also signify an inhibition on the testicular somatic index, which may be caused by hyperactivation of the hypophysical–adrenocortical axis as a result of disease or stressful conditions, as reported by Jana et al. [25]. On the other hand, sperm counts in the testis and epididymis are reduced by the direct suppression of testosterone production during stress conditions due to excessive levels of adrenocorticotropic and corticosterone hormones [26].

### 2.2. Testicular Apoptosis

Cell death can occur in the male reproductive system especially the testis and epididymis through necrosis, autophagy, entosis, and apoptosis [27]. Necrosis is triggered by infection, toxins, or trauma, which normally prompts an immune response. Similarly, apoptosis is a cellular program that does not cause cell lysis and cannot initiate an inflammatory reaction [28]. It is chararacterized by the conversion of procaspase 8 to caspase 8 caused by an increased Fas ligand, which stimulates caspase 3 from procaspase 3 and thereafter, in conjunction with caspase 9, results in apoptosis. Environmental agents, cell injury, or stress are the major stimulants of testicular apoptosis. Therefore, apoptosis is a response to deprivation of survival factors, including testosterone, activation by ligated death factors, and exposure to environmental stimuli such as radiation, chemotherapeutic drugs, and radical oxygen species (ROS) that activate cascade reactions of caspases [29,30,31,32].

Sakkas and El-Fakahany [33] reported that ROS initiates a cascade of reactions that ultimately trigger apoptosis. Furthermore, some studies have been reported in rats, mice, and humans in which testicular apoptosis was implicated in subfertility and or infertility [34,35,36,37,38]. Venkatesan and Sadiq [39] reported that the administration of mercury chloride in male rats showed increases in the expression of Bax and caspase-3 and a marked decrease of Bcl-2 level relative to the control group.

### 2.3. Testicular Reactive Oxygen Species and Oxidation Stress

Oxidative stress is described as a situation in which a system has an imbalance between oxidation and reduction reactions, leading to the generation of excess oxidants or molecules that accept an electron from another reactant [40]. Oxidative stress occurs when the production of potentially destructive reactive oxygen species (ROS) exceeds the body’s own natural antioxidant defenses, resulting in cellular damage [41]. It is also a common pathology seen in approximately half of all infertile men in which several environmental pollutants have also been linked with testicular oxidative stress [42,43]. There has been increasing evidence indicating that oxidative stress is increased in metabolic diseases such as diabetes and obesity due to the overproduction of ROS and decreased efficiency of antioxidant defenses; this becomes worsened as the disease progresses [44]. For example, Suleiman et al. [45] reported that the administration of a high fat-diet (HFD) to rats for 12 weeks caused increases in the malondialdehyde (MDA) levels and decreases in superoxide dismutase (SOD), catalase (CAT), glutathione reductase (GR), glutathione peroxidase (GPx), and glutathione-S-transferase (GST) activities and a decrease in reduced glutathione (GSH) level in the testis and epididymis. Similarly, diabetic rats induced with a single dose of 40 mg/kg bw streptozotocin (STZ) intraperitoneally had an increase in MDA level and decreases in antioxidant enzymes activities and total antioxidant capacity in the testis [46].

Consequently, some authors have reported an increase in MDA level and a decrease in the SOD activity in galatose-induced oxidative stress in the testis of rats with accompanying decreases in the sperm counts and testicular weights [47]. Similarly, lead increases MDA and nitrite levels, and it decreases GSH content and CAT activity in the testis of rats [48]. After treatment with 1,1,1-trichloro-2,2-bis(4-chlorophenyl) ethane (p,p′-DDT) for 10 days in rats, increases in MDA and H_2_O_2_ levels as well as GSSG activity were seen alongside decreases in SOD, CAT, GR, GPx, GST activities, and GSH level [49].

### 2.4. Testicular Inflammation

The testis is known for its role in the immune system; aside from playing a major part in the maintenance of spermatogenesis, it also possesses the immune–testicular barrier, which justifies the increased CD8+/CD4+ ratio in the testis to that in the circulation [50]. The testis also has lots of immune cells, such as macrophages, mast cells, and natural killer cells in the interstitial and peritubular compartment; however, the number of lymphocytes in the testis is relatively small. In addition to their impact on testis-specific functions, macrophages in the testis are considered as potential effector cells in the first line of host defense. They express major histocompatibility complex class II (MHC II) molecules essential for antigen presentation to CD4 T cells and releases proinflammatory cytokines such as interleukin-1 (IL-1), IL-6, and tumor necrosis factor- (TNF-α). However, several studies indicate that macrophages in the normal adult testis mainly exert anti-inflammatory activities [51,52,53].

Some authors have established that illnesses and systemic infections have detrimental effects on the male reproductive system. For instance, bacterial lipopolysaccharide, which is used in animals to induce systemic and localize inflammation, have caused decreases in androgens, pro-inflammatory cytokines, IL-1β, and TNF-α [53,54]. 

Heavy metals have also caused inflammation in the testis of experimental animals. Almeer et al. [55] reported that the administration of mercury chloride (HgCl_2_) in rats resulted in the release of interleukin-1β and TNF-α. Similarly, the administration of lead acetate in rats showed significant increases in IL-1β, TNF-α, and monocyte chemoattractant protein-1 (MCP-1) in the testis. Metabolic diseases such as diabetes [56,57] and obesity [58,59] have also been implicated in severe inflammation in rats and mice.

**Figure 1:**
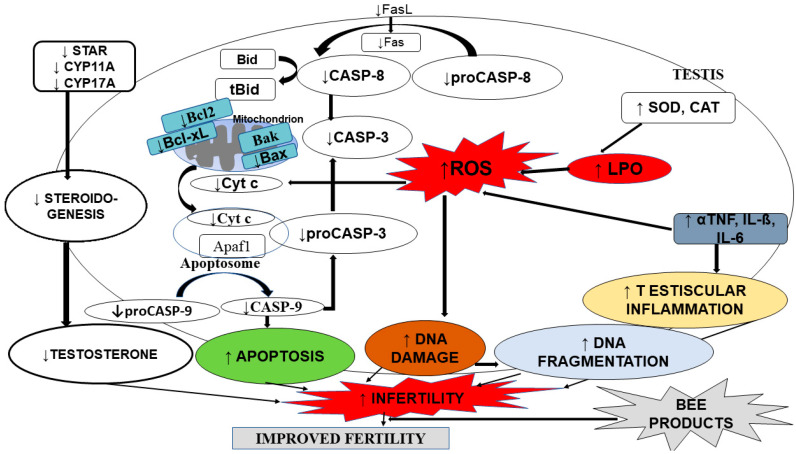
Factors involved in testicular impairment (adapted from [60]). Apaf1: Apoptotic protease activating factor 1; Bak: Bcl2 Antagonist/Killer;Bax: Bcl-2-like protein 4; Bcl2: B-cell lymphoma 2; Bid: Bax-like BH3 protein; CASP-3: Caspase 3; CASP-9: Caspase 9; CASP-8: Caspase 8; CAT: Catalase; CYP11A: Cytochrome P450 11A; CYP17A: Cytochrome P450 17A; Cyt c: Cytochrome c; DNA: Deoxyribonucleic acid; FasL: Fas ligand; IL-6: Interleukin 6; IL-β: Interleukin β; LPO: Lipid peroxidation; proCASP 3: proCaspase 3; proCASP 8: pro Caspase 8; proCASP 9: pro Caspase 9; SOD: Superoxide dismutase; StAR: Steroidogenic acute regulatory protein; αTNF: α Tumor necrotic factor.

These factors involved in the male reproductive impairment are summarized in Figure 1.

## 3. Composition of Bee Products

The composition of the various bee products is highlighted below. Honey is a sweet substance produced by bees and stored in beehives [61]. It is formed from nectar collected from various flowers. Honey is consumed as a nutrient with a traditional belief to enhance general health including fertility status. It is acidic (pH 3.2–4.5) and composed of sugars (fructose, glucose, sucrose, maltose contents, and glucose), water, carbohydrates, nitrogenous substances and elements, proteins, organic acids (acetic, butyric, citric, formic, gluconic, lactic, malic, pyroglutamic, and succinic), polyphenols, and cyclitols as well as antioxidants [6,62,63].

Propolis is a combination of beeswax, tree resins, honey, and enzymes made by bees to protect the hive from external threats, such as bacteria or viruses or storage of honey and bee bread. It contains strong antiviral, antifungal, anti-inflammatory, and antibacterial properties. Propolis is a natural resinous substance collected by bees from parts of plants, buds, and exudates. Bees use it as a sealer for their hives and, more importantly, to prevent the decomposition of creatures that have been killed by bees after an invasion of the hive. It is slightly acidic (pH 4.35–6.92) and composed of water (3–8%), carbohydrates (25%) (xylose, galactose, mannose, glucuronic acid, lactose, maltose, melibiose, erytritol, xylitol, inositol), amino acids (25%) (aspartic acid, glutamic acid [7], serine, glycine, histidine, arginine, threonine, alanine, proline, tyrosine, valine, methionine, isoleucine, leucine, phenylalanine, lysine, tryptophan) [64], vitamins B1 and B2, flavonoids, terponoids, phenolics, and quercetin [65].

Royal jelly is a secretion of the mandibular and hypopharyngeal glands of young worker nurse honeybees; it is a milky-white or yellowish creamy and acidic material with a slightly pungent odor and taste. The young larvae, brood of workers, and drones depend on it as food temporarily, but it is the sole food of the queen bee for both her larval and adult life. It is acidic (pH 3.4–4.5) and composed of water (60–70%), lipids (3–8)%, 10-hydroxy-2-decenoic acid (>1.4)%, protein (9–18)%, fructose (3–13)%, glucose (4–8)%, sucrose (0.5–2.0)%, and ash (0.8–3.0)%. It also contains vitamins (riboflavin, thiamine, niacin and folic acid), its major elements are potassium, phosphorus, sulfur, sodium, calcium, aluminum, magnesium, zinc, iron, copper, and manganese, but there are trace amounts (0.01–1 mg/100 g) of nickel, chromium, tin, tungsten, antimony, titanium, and bismuth [66].

The survival of bees largely depends on pollen grains as their source of proteins. Its composition includes proteins, amino acids, carbohydrates, lipids, fatty acids, phenolic compounds, enzymes, coenzymes, vitamins, and bioelements [6,62,63]. These pollens are collected from flowers and stored inside the cells separately from the nectar cells of the beehive. Bee pollen is made up of 7–17% water, 36–37% carbohydrate (fructose and glucose), 20–23% proteins (methionine, lysine, threonine, histidine, leucine, isoleucine, valine, phenylalanine, and tryptophan), 5.1% fat, 2.2–3% ash content, phenolics compounds 1.6% (flavonoids, leukotrienes, catechins, and phenolic acids) [67].

Bee brood is considered to be the eggs, larvae, and pupae of honeybees, which develops within a bee hive. It is composed of water (76.8%), proteins (9.4%), amino acids (7.9%), lipids (4.7%), fatty acids (4.0%), carbohydrates (8%), fiber content (0.5%), and ash (0.8%) [66].

Apilarnil consists of mainly bee drone larvae and trace amounts of royal jelly, bee bread, honey, and propolis. Usually, it is not utilized, and honeycombs with apilarnil are cut and discarded by beekeepers. It has biological properties which include antiviral, immune system enhancer, and anabolic stimulator, and it also increases appetite, body energy, vitality, and regenerative power. It is also very rich in androgenic hormones, so it stimulates spermatogenesis in men [68]. It contains dry matter (25–35%), proteins (9–12%), carbohydrates (6–10%), lipids (5–8%), ash (2%), and unidentified substances (3%) [68].

Honeybees create beeswax to build their hive or pots and store both honey and pollen. It is commonly used in cosmetic products. However, the strength, flexibility, and water-proofing qualities of beeswax have made it an excellent material for polishes, finishes, and waxes that preserve, add shine, and generally enhance products coated with it. Beeswax stability also makes it an excellent wax for addition to cosmetics and skin products. Historically, beeswax was an excellent material for making molds for castings; indeed, even today, we have artifacts over 3000 years old that were produced by the lost-wax process. It is composed of monoesters, diesters, hydroxylated esters, hydrocarbons, and free fatty acids [4].

Bee venom is produced by the female worker bees. It is usually delivered directly from a bee sting. The treatment of some diseases can be done by the administration of the bee sting to the skin through a stainless-steel micro mesh, which allows the venom to enter the skin but prevents the stinger from being attached to the skin. It comprises of peptides such as melittin and apamin, mast cell degranulating (MCD) peptide, adolapin, tertiapin, secapin, melittin F, and cardiopep as well as enzymes such as phospholipase A2 (PLA2), phospholipase B (PLB), hyaluronidase (cytotoxicity), phosphatase, and α-glucosidase (nontoxic) [69].

## 4. Role of Bee Products in Male Reproductive Impairment

There are a lot of studies of bee products used in ameliorating male reproductive impairment. Table 1, Table 2, Table 3, Table 4, Table 5 and Table 6 show the summary of various effects of bee products on the male reproductive system in various animal models and human.

### 4.1. Effects of Bee Pollen on Male Reproductive Parameters

The administration of 100 mg/kg bw/day of bee pollen on streptozotocin (STZ)-induced diabetic rats for 4 weeks caused significant increases in testis weight, testosterone, LH, and FSH as well as sperm count, motility, and viability, which is suggested partly by scavenging toxic and mutagenic electrophiles and free radicals/modification of antioxidant pathways due to the presence of flavonoids [12]. Algerian bee pollen (100 mg/kg bw) administered for 15 days showed an increase in spermatogenesis and a decline in Sertoli cells destruction by lowering lipids, and it also showed anti-inflammatory and protective effects against testis cell injury due to the potentiated synthesis of proteins. Similarly, 60 mg/animal/day of Turkish bee pollen over a 30-day period showed increases in testosterone level and sperm counts in a rats model via its antioxidant activity [70].

Furthermore, the Indian bee pollen of 100 mg/kg/bw caused a decrease in MDA levels, while there were increases in SOD, GR, GPx, GST, CAT, and GSH in rifampicin and isoniazid-induced toxicity in rats through its antioxidant activity. In addition, lead-induced rats treated with 100 mg/kg bw of Algerian bee pollen showed an increase in spermatogenesis and a decline in the destruction of Sertoli cells (Table 1).

**Table 1:** Effects of bee products on male reproductive parameters.

s/*n*	Bee Products	Dose/Duration of Treatment	Substance Used to Induce Stress	Animal Model Used	Route of Administration	Standard Drug	Effect on Reproductive Function Parameters	Possible Molecular Mechanisms	References
1.	Bee pollen (Egypt)	100 mg/kg bw/day for 4 weeks	Streptozotocin (STZ)-injection (single dose)	Rats	i.p	-	↑ Testis weight, testosterone, LH, FSH, sperm count, motility and viability, ↓ MDA, ↑ (SOD, GR, GPx, GST, CAT, and GSH)	Act by scavenging toxic and mutagenic electrophiles and free radicals/modification of antioxidant pathways due to presence of flavonoids	[12]
2.	Bee pollen (India)	100 mg/kg bw	Rifampicin 100 mg/kg bw/day and isoniazid 50 mg/kg bw/day	Rats	Oral	-	↓ MDA, ↑ (SOD, GR, GPx, GST, CAT, and GSH)	Presence of bioactive elements (caffeic acid phenethyl ester, myricetin, kaempherol, isoquercetin, and flavonoids) convert the reactive free radicals to inactive products	[71]
3.	Bee pollen (Algeria)	100 mg/kg bw for 15 days	30 mg mg/kg bw of lead acetate	Rats	Oral	-	↑ Spermatogenesis and ↓ Sertoli cells destruction	Acts by lowering lipid, anti-inflammatory, and protective effect against testis cell injury due to potentiated synthesis of proteins	[72]
4.	Bee pollen (Turkey)	60 mg/per animal (30-day)		Rats	Oral		↑ Testosterone level and sperm counts	Beneficial effects	[70]

bw: body weight; CAT: catalase; FSH: follicle-stimulating hormone; GPx: glutathione peroxidase; GR: glutathione reductase; GSH: glutathione; GST: glutathione-S-transferase; LH: leutinizing hormone; LPO: lipid peroxidation; MDA: malondialdehyde; SOD: superoxide dismutase; STZ: streptozotocin.

### 4.2. Effects of Bee Venom on Male Reproductive Parameters

Few studies have been reported on the effects of bee venom on the testicular damage; Egyptian bee venom at doses of 0.1, 0.2, and 0.3 mg/rabbit twice weekly administered over 20 weeks showed increases in TAC, GST, GSH, testosterone spermatogenesis, and fertility. These may be due to the stimulation of the pituitary gland to release the adrenocorticotropic hormone, which causes release of the sex hormones such as testosterone in blood circulation, which have significant effects on spermatogenesis and fertility [73]. In a related study carried out in mice treated with Iraqi bee sting, it provided protection and the maintenance of some sexual efficiency parameters via its ability to release cortisol that inhibits Sertoli cells from releasing activin-B, which normally stimulates spermatogonia to induce mitosis to form spermatocytes [10] (Table 2).

**Table 2:** Effects of bee venom and bee wax on male reproductive parameters.

s/*n*	Bee Products	Dose/Duration of Treatment	Substance used to Induce Stress	Animal Model Used	Route of Administration	Standard Drug	Effect on Reproductive Function Parameters	Possible Molecular Mechanisms	References
1.	Bee Venom (Egypt)	0.1 (G1), 0.2 (G2) and 0.3 (G3) mg/rabbit twice weekly over 20 wks	High temperature	Rabbits	Intravenous injection	-	↑ TAC, GST, GSH, IgA, IgM, Testosterone, spermatogenesis and fertility	These effects could be attributed to pituitary gland stimulation to release the adrenocorticotropic hormone, which causes release of the sex hormones such as testosterone in blood circulation, which has significant effects on spermatogenesis and fertility	[73]
2.	Bee Venom (Iraq)	155 stings	hydrogen peroxide	Mice	Stings	-	Protection and maintenance of some sexual efficiency parameters	Cortisol inhibits Sertoli cells from releasing activin-B, which normally stimulates spermatogonia to induce mitosis to form spermatocytes	[10]
3.	Bee venom (Romania)	700 μg BV/kg		Rats	Injection	-	↓ Testicular weight and Sertoli cells, ↑diameter in seminiferous tubules	Mellitin interacts with the proteins in tight junctions between the adjacent Sertoli cells	[13]
4.	Bee wax (USA)	15 mg bees wax pellet containing 3.0 mg		Mice	Injection	-	Differential testicular response to photoperiod	Post-pineal mechanism	[74]

TAC: total antioxidant capacity; GST: glutathione-S-transferase, and GSH: glutathione; IgA: immunoglubolin A; IgM: immunoglubolin M.

### 4.3. Effects of Honey on Male Reproductive Parameters

Studies carried out in Nigeria revealed that 100, 200, and 400 mg/kg of honey administered on rats and 2.5, 5, and 7.5 mg/kg of testosterone i.p. showed increases in sperm count; this might be due to the fact that chrysin (5,7-dihydroxyflavone) blocked the conversion of androgens into estrogens with a consequent increase in testosterone [75]. On the other hand, 70 g of Iranian honey supplementation administered on humans for 8 weeks showed significantly less elevation in seminal IL-1β, IL-6, IL-8, TNF-α, ROS, and MDA levels and increases in seminal SOD, catalase, and TAC concentrations through its antioxidant, anti-inflammatory, and anti-apoptotic properties due to the presence of phenols and flavanoids [76]. Similarly, 1.0 mL/100 g body weight of honey administered in nicotine-induced old rats showed increases in the fertility of juvenile male rats by increasing sperm motility and the number of morphologically normal sperm; however, the exact mechanisms require further study [77].

In addition, 0.05 mL of honey administered for 4 weeks showed diminished degenerative changes of seminiferous tubules and increased plasma levels of testosterone significantly in CCL-induced rats via reduction of the elevated levels of free radicals and an increased antioxidant defense system [78]. Furthermore, rats treated with 1.0 mL/100 g of Egyptian honey for 60 days showed significant increases in sperm count and the number of sperm with normal morphology, the honey acted as a physiologic modulator of spermatogenic cells proliferation, which influence the cell cycle of the seminiferous epithelium; thereby, it increases spermatogenesis [14]. Similarly, studies carried out in Iran by Hadi and Mohammed [79,80] revealed that 10% of honey (1 mL of honey and 9 mL of IVF culture medium) with doses of 1.2 and 1.8 g/kg bw enhances sperm motility, increases testosterone, FSH, and LH hormones as well as diameters of seminiferous tubules; this might be a result of the antioxidant properties of honey.

Conversely, 1.2 g/kg of Malaysian honey showed increases in the percentages of rats achieving intromission, ejaculation, mating, and fertility indexes as well as increases in testis, epididymis weights, percentages of abnormal spermatozoa, and sperm motility; in this case, the mechanism through which honey acts is by its counteraction on oxidative stress within penile tissues via its antioxidant property due to the possession of phenols [81,82].

Likewise, 0.2, 1.2, and 2.4 g/kg^−1^ of Malaysian honey administered for 4 weeks in rats revealed increases in epididymal sperm count without affecting spermatid count and reproductive hormones [83,84]. Furthermore, 1, 2, and 2.5 mL of Nigerian honey administered to rats for 21 days improved the sperm quality and spermatogenesis rate, and there was no sign of degeneration or cellular loss in the testicular histoarchitecture. It is imperative to note that the presence of zinc in honey and its accumulation in the testis during early spermatogenesis may be important in DNA synthesis and regulate spematogonial proliferation [85]. In other similar studies, 1 mL/100 g of bw of Nigerian honey administered for 65 days increases the sperm count and sperm motility, and it also improves the sperm morphology through the reduction of lipid peroxidation and oxidative stress on the sperm cells by reactive oxygen species such as superoxide and hydrogen peroxide. The authors of [86,87,88] revealed that rats treated with 100 mg/kg bw of Nigerian honey for 35 days had improvements in sperm motility, viability, morphology, counts, FSH, LH, and testosterone. The rats treated with 5% Palestinian honey for 20 days induced spermatogenesis in rats by increasing epididymal sperm count, relative weight of the epididymis, SDH activity, and reducing LDH activity; however, the mechanisms require further study [89].

Saudi Arabian honey (20 mg/kg bw/day) ameliorates octylphenol toxic effects and reduces the histopathological stress toxicity on the testis in rats; also, the combined administration of honey and royal jelly reduces sperm abnormality and chromosomal aberrations as well as ameliorates GSH and MDA in cyclophosphamide toxicity in mice; therefore, the presence of CAPE served as a protective agent against chemotherapy-induced oxidative stress [9,90]. The honey drone milk is a product that is secreted by honey bees through their hypopharyngeal and mandibular glands; thus, the Hungarian honey drone milk (110 mg/kg/day) increases the relative weights of the androgen-dependent organs and the plasma testosterone level in castrated rats and then increases the tissue mRNA and protein level of SLAP (Spot14-like androgen-inducible protein). This was done through the scavenging of free radicals by polyphenols before they can interact with DNA [91], while 70 g of honey supplement administered to humans for 8 weeks in Iran increases seminal IL-1b, IL-6, IL-8, TNF-α, ROS, and MDA levels and significantly decreases the levels of seminal SOD, catalase. Kelulut honey 2.0 g/kg weight administered 28 days to diabetic rats revealed significant increases in SOD activity and GSH level as well as significant decreases in protein carbonyl and MDA levels in sperm and testis, whereas the histology of the epididymis showed a decrease in spermatozoa and spermatogenic cells density in the testis of the diabetic group [11] (Table 3).

**Table 3:** Effects of honey on male reproductive parameters.

s/*n*	Bee Products	Dose/Duration of Treatment	Substance used to Induce stress	Animal Model Used	Route of Administration	Standard Drug	Effect on Reproductive Function Parameters	Possible Molecular Mechanisms	References
1.	Honey (Nigeria)	100, 200, and 400 mg/kg	-	Rat	Oral	2.5, 5, and 7.5 mg/kg of testosterone i.p	↑ Sperm count	Chrysin (5,7-dihydroxyflavone) blocked the conversion of androgens into oestrogens with a consequent increase in testosterone	[75]
2.	Honey (Egypt)	0.05 mL (4 weeks)	5 mL/kg of 0.3% CCL 4 daily subcutaneously (4 Weeks)	Mice	Oral	-	↓ Degenerative changes of seminiferous tubules and ↑ plasma levels of testosterone significantly	Via reduction of the elevated levels of free radicals and increase in the antioxidant defense system	[78]
3.	Honey (Malaysia gelam honey)	1.0 mL/100 g (60 days)	-	Rats	Oral	-	↑ Sperm count and number of sperm with normal morphology	Acts as a physiologic modulator of spermatogenic cells proliferation, which influence the cell cycle of the seminiferous epithelium thus, ↑ spermatogenesis	[14]
4.	Honey (Malaysia)	1.2 g/kg bw/daily	Cigarette 8 min 3 times/day	Rats	Oral	-	↑ Intromission and ejaculation, mating, and fertility indexes	Acts as a physiologic modulator of spermatogenic cells proliferation, which influence the cell cycle of the seminiferous epithelium and thus increase spermatogenesis	[82]
5.	Honey (Malaysia)	1.2 g kg^−1^ bw daily (21 days)	Prenatal restraint stress (three times per day) from day 11 of pregnancy until delivery	Rats	Oral	-	↑ Testis and epididymis weights as well as improved the percentages of abnormal spermatozoa and sperm motility	Acts partly by its counteraction on oxidative stress within penile tissues via its antioxidant property	[81]
6.	Honey (Malaysian honey)	0.2, 1.2, and 2.4 g kg^−1^ (4 weeks)	-	Rats	Oral	-	↑ Epididymal sperm count without affecting spermatid count and reproductive hormones	Due to its one or more constituents that could protect germ cells against oxidative stress. This might have further enhanced spermiogenesis	[83]
7.	Honey (Nigeria)	1, 2, and 2.5 mL of honey daily for 21 days	-	Rats	Oral	0.3 mL FSH drug for 6 days	Improves the sperm quality and spermatogenesis rate and no sign of degeneration or cellular loss in the testicular histoarchitecture	Suggestive of zinc accumulating in the testis during early spermatogenesis, and important in DNA synthesis and the regulation of spematogonial proliferation	[85]
8.	Honey (Nigeria)	1 mL of honey per 100 g of bw (65 days)	-	Rat	Oral	Manix capsules (6220 mg/100 mL of drug solution)	↑ Sperm count, sperm motility, and improves sperm morphology	↓ Lipid peroxidation and oxidative stress on the sperm cells by reactive oxygen species such as super oxide, hydrogen peroxide	[87]
9.	Honey (Nigeria)	(100 mg/kg bw) (35 days)	Nicotine (1.0 mg/kg bwt)	Rats	Oral	-	↑ Sperm motility, viability, morphology, counts, FSH, LH, and testosterone	Mediated by its counteraction on oxidative stress	[88]
10.	Honey supplements (Iran)	70 g (8 weeks)	8 weeks of intensive cycling training	Humans	Oral	-	↓ Seminal interleukin (IL)- 1 b, IL-6, IL-8, tumor necrosis factor (TNF)-α, ROS, MDA, ↑ Levels of seminal SOD and catalase	↓ Seminal plasma cytokines and oxidative stress biomarkers as well as increasing seminal antioxidant levels	[76]
11.	Honey (Palestinian Honey)	5% honey for 20 days	-	Rats	Oral	-	Induces spermatogenesis in rats by ↑ epididymal sperm count, relative weight of the epididymis, SDH activity, and ↓ LDH activity	Needs further experiments to establish mechanism	[89]
12.	Honey (Saudi Arabia)	20 mg/kg body weight/day) for 4 weeks	Octylphenol (0.1 and 1.0 mg kg_1 bw)	Rats	Oral	-	Ameliorates toxic effects and ↓ histopathological stress toxicity	Further studies required	[9]
13.	Honey (Taulang) (Malaysia)	0.2, 1.2, or 2.4 g/kg/day of honey for 28 days	-	Rats	Oral	-	↑ Sperm counts significantly.	Further studies required	[84]
14.	Honey bee and pollen grains (Saudi Arabia)	(1 g/kg) 2 weeks	Cyclophosphamide (10 mg/kg) i.p	Mice	Oral	-	↓ Sperm abnormality, chromosomal aberrations, ameliorates GSH and MDA	Presence of CAPE as protective agent against chemotherapy-induced oxidative stress	[90]
15.	Honey bee Drone milk (Hungary)	110 mg/kg/day	-	Castrated Rats	Oral	-	↑ Relative weights of the androgen-dependent organs and the plasma testosterone level in castrated rats and tissue mRNA and protein level of SLAP	Scavenging of free radicals by polyphenols before free radicals can interact with DNA	[91]
16.	Honey (Iran)	10% of honey	-	Mice	IVF	-	Enhances sperm motility and pregnancy rate of female mice	Antioxidant activity	[79]
17.	Honey (Gelam) (Malaysia)	1.0 mL/100 g bw	Nicotine (N) group were intraperitoneally (i.p.) injected with 5.0 mg/kg	Rats (4–5 weeks old)	Intra peritoneal		↑ Fertility of juvenile male rats by increasing sperm motility and number of morphologically normal sperm	Further study required	[77]

ADP: adenosine diphosphate; AlCl_3_: aluminum chloride; bw: body weight; CAT: catalase; CCL: carbon tetra chloride; DHEA-S: dehydroepiandrosterone sulfate; DNA: deoxyribonucleic acid; FeSO_4_: ferrous sulfate; FSH: follicle-stimulating hormone; GPx: glutathione peroxidase; GR: glutathione reductase; GSH: glutathione; GST: glutathione-S-transferase; HSP 70: heat shock protein 70; i.p: intraperitoneal; IL: interleukin; IVF: in vitro fertilization; LDH: lactate dehydrogenase; LH: leutinizing hormone; L-NAME: N^ω^-nitro-l-arginine methyl ester; LPO: lipid peroxidation; MDA: malondialdehyde; NF-κB: nuclear factor kappa B; p.o: per os; PCNA: proliferating cell nuclear antigen; PON1: paraoxonase 1; ROS: reactive oxygen species; SLAP: Spot14-like androgen-inducible protein; SOD: superoxide dismutase; STZ: streptozotocin; TAC: total antioxidant capacity; TNF: tumor necrosis factor.

### 4.4. Effects of Propolis on Male Reproductive Parameters

Iraqi propolis of 200 mg/kg bw decreases the sperm concentration, sperm motility, rate of viability, and normal sperms as well as decreases the weights of testes, epididymis, prostate gland, seminal vesicles, serum testosterone, FSH, and LH levels with a significant increase in sperm abnormalities in acrylamide-induced toxicity in rats through the antioxidative effectiveness of propolis mainly by its flavonoids and phenolic content [92]. Egyptian propolis extract (50 mg/kg bw) decreases LPO levels and normalizes CAT, SOD, GPx, and GST activities, while GSH content was increased in testicular tissue in chlorpyrifos-induced toxicity in rats. The protective effect can be due to scavenging MDA molecules by propolis active ingredients or inhibition of mitochondrial and cytosolic lipoperoxidation chain reactions [93]. Egyptian propolis of 200 mg/kg p.o. for 3 weeks decreases testicular oxidative stress, inflammatory, and apoptotic markers in doxorubicin-induced toxicity in rats due to its possession of phenolic compounds [15]. Egyptian propolis (50 mg/kg bw/day extract decreases dead and abnormal sperm and TBARS, and it increases testosterone, GSH, 17-ketosteroid reductase, CAT, and GST in aluminum chloride-induced toxicity in rats through its antioxidant properties [94].

Turkish propolis (100 mg/kg/day) prevented the rise in malondialdehyde, xanthine oxidase levels, and HSP-70 expression and improved testicular morphology and JTBS in methotrexate-induced toxicity in rats through scavenging free radicals and thereby protected against lipid peroxidation [95]. Similarly, the combination of Turkish propolis (200 mg/kg/days, gavage) and pollen (100 mg/kg/days, by gavage) decreases levels of TOS, NF-κB, and MDA using L-NAME (40 mg/kg, i.p.) for induction of hypertension in rats; this was done by inhibiting the functioning of inflammatory pathways [96]. The in vitro study carried out by [97] shows that Chilean propolis protects sperm membrane from the deleterious action of oxidative attack, reducing TBARS formation and LDH release by exhibiting a strong antioxidant activity of propolis. Similarly, 1 uL of Czech Republican propolis maintains sperm motility and improves the total mitochondrial respiratory efficiency in human spermatozoa through its antioxidant properties [98]. Egyptian propolis (50 mg/kg bw/day) improves the structure of seminiferous tubules, and their lumens were full of bundles of sperms. In addition, all the parameters of seminiferous tubules and total numbers of Sertoli cells, round spermatids, daily sperm production, and Leydig cells were ameliorated through decreases in the levels of free radicals and lactate dehydrogenase as a result of the presence of flavonoids [99]. Egyptian propolis administered to rabbits at 100, 200, and 300 mg/kg bw/day, respectively for two weeks (one week before and after mating) for five consecutive times shows that the bunnies belong to rabbits treated with bee propolis, which shows the best improvement for all the studied traits due its antioxidant nutrients, including vitamins, minerals, phenolic constituents, and enzymes [100].

Egyptian propolis (50 mg/kg bw) revealed significant decreases in CAT, SOD, GPx, and GST in chlorpyrifos-induced toxicity [93]. Rats treated with 3, 6, and 10 mg/kg/day of green Brazallian propolis show higher sperm production and greater epithelium height of the epididymis initial segment and no induction of oxidative stress, and the exact mechanism is still under investigation [101]. The co-administration of Turkish propolis (200 mg/kg/days, gavage) and pollen (100 mg/kg/days, by gavage) that lasted 14 of 28 days showed decreases in TOS, NF-κB, MDA, TAS levels, PON1, and CAT activities in testis tissue; it acted through its protective effect of antioxidant mechanisms [96]. Furthermore, Malaysian propolis (300 mg/kg bw) administered on streptozotocin-induced rats caused increases in testosterone level, steroidogenic, and sperm parameters by increasing penile cGMP and serum testosterone levels due to the presence of phenols [102] (Table 4).

**Table 4:** Effects of propolis on male reproductive parameters.

s/*n*	Bee Products	Dose/Duration of Treatment	Substance Used to Induce Stress	Animal Model Used	Route of administration	Standard Drug	Effect on Reproductive Function Parameters	Possible Molecular Mechanisms	References
1.	Propolis (Iraq)	200 mg/kg bw (4 weeks)	Acrylamide (150 mg/kg BW)	Rats	Oral	-	↓ Sperm concentration, sperm motility, rate of viability, normal sperms, weights of testes, epididymis, prostate gland, seminal vesicles, serum testosterone, FSH, LH levels with significant ↑ sperm abnormalities	Anti-oxidative effectiveness of propolis mainly via its flavonoids and phenolic content	[92]
2.	Propolis (Egypt)	50 mg/kg bw extract (70 days)	Chlorpyrifos (9 mg/kg) (insecticide)		Oral	-	↓ LPO level, normalized CAT, SOD, GPx, and GST activities, ↑ GSH content in testicular tissue	Protective effect can be due to scavenging MDA molecules by propolis active ingredients or inhibition of mitochondrial and cytosolic lipoperoxidation chain reactions	[93]
3.	Propolis (Egypt)	Propolis extract (200 mg kg 1; p.o.) for 3 weeks	Doxorubicin 18 mg kg 1 total cumulative dose of Dox i.p.	Rats	Intraperitoneal	-	↓ Testicular oxidative stress, inflammatory and apoptotic markers	Tumor necrosis factor-related apoptosis inducing ligand via phenolic compounds	[15]
4.	Propolis (Egypt)	50 mg propolis/kg bw/day	Aluminium chloride 34 mg AlCl_3_/kg bw (70 days)	Rats	Oral	-	↓ Dead and abnormal sperm and TBARS, and ↑ testosterone, GSH, 17-ketosteroid reductase, CAT, and GST	Antioxidant property of propolis	[94]
5.	Propolis (Turkey)	100 mg/kg/day (oral gavage) (15 days)	Methotrexate (20 mg/kg)	Rats	Oral	-	↓ Malondialdehyde, xanthine oxidase levels, and HSP-70 expression and improves testicular morphology and JTBS	Scavenging free radicals and thereby protection against lipid peroxidation	[95]
6.	Propolis (Balikesir, Turkey)	Propolis (200 mg/kg/days, gavage) and pollen (100 mg/kg/days	L-NAME (40 mg/kg, i.p.) for induction of hypertension	Rats	Oral	-	↓ Levels of TOS, NF-κB, and MDA	Inhibiting the functioning of inflammatory pathways	[96]
7.	Propolis (Chilean propolis)	-	benzo[a]pyrene, hydrogen peroxide (H_2_O_2_) and hydrogen peroxide in combination with adenosine 5 V-diphosphate (ADP) and ferrous sulfate (FeSO_4_)	Human spermatozoa	In vitro	-	Protects sperm membrane from the deleterious action of oxidative attack, reducing TBARS formation and LDH release	Exhibited a strong antioxidant activity	[97]
8.	Propolis (Czech Republic)	(1 uL) 10 participants		Human spermatozoa (0.1 mL of fresh ejaculate)	In vitro	-	Maintains sperm motility and improves the total mitochondrial respiratory efficiency	Antioxidant property	[98]
9.	Propolis (Egypt)	50 mg/kg bw/day	-	Rats	Oral	Intraperitoneal injection of genta micin (5 mg/kg bw/day)	Improves structure of seminiferous tubules and ↑ daily sperm production	↓ Level of free radicals and lactate dehydrogenase	[99]
10.	Propolis (Egypt)	100, 200, and 300 mg/kg bw/day, respectively for two weeks (one week before and after mating) for five consecutive times	-	New Zealand White (NZW) rabbit		-	Improves all studied traits	Substantial levels of antioxidant nutrients, including vitamins, minerals, phenolic constituents, and enzymes	[100]
11.	Propolis (green brazallian propolis)	3, 6, and 10 mg/kg/day (56 days)	-	Rats	Oral	-	↑ Sperm production and greater epithelium height of the epididymis initial segment and no induction of oxidative stress	Mechanism still under investigation	[101]
12.	Propolis (Egypt)	50 mg kg/bw (4 weeks)	Paclitaxel 5 mg/kg/bw	Rats	Oral	-	↑ Sperm count, motility, viability, and sperm morphology	Scavenging the free radicals and enhancing the antioxidant activities	[8]
13.	Propolis (India)	400 mg/kg bw (5 days a week for 4 weeks)	Mitomycin C (2, 4, and 8 mg/kg bodyweight, single dose) (i.p)	Mice	Oral	-	↓ Oxidative stress and DNA damage, ↑ testicular testosterone and inhibin B	Strong antioxidant activity	[103]
14.	Propolis + Bee pollen (Turkey)	Propolis (200 mg/kg/day) and pollen (100 mg/kg/day) the last 14 of 28 days	N(ω)-nitro-L-arginine methyl ester (L-NAME) (40 mg/kg, i.p.)	Rats	Oral		↓ TOS, NF-κB, MDA, TAS levels, PON1, and CAT activities in the testis tissue	Protective effect of antioxidant mechanisms against oxidative mechanisms on the reproductive system	[96]
15.	Propolis (Malaysia)	Propolis (300 mg/kg bw for 4 weeks	streptozotocin (60 mg/kg bw	Rats	Oral	Metformin (300 mg/kg/day	↑ Testosterone level, steroidogenic and sperm parameters	↑ In penile cGMP and serum testosterone levels due to presence of phenols	[102]

ADP: adenosine diphosphate; AlCl_3_: aluminum chloride; bw: body weight; CAT: catalase; cGMP: cyclic guanidine monophosphate; CCL: carbon tetra chloride; DHEA-S: dehydroepiandrosterone sulfate; DNA: deoxyribonucleic acid; FeSO_4_: ferrous sulfate; FSH: follicle-stimulating hormone; GPx: glutathione peroxidase; GR: glutathione reductase; GSH: glutathione; GST: glutathione-S-transferase; HSP 70: heat shock protein 70; i.p: intraperitoneal; IL: interleukin; IVF: in vitro fertilization; LDH: lactate dehydrogenase; LH: leutinizing hormone; L-NAME: N^ω^-nitro-l-arginine methyl ester; LPO: lipid peroxidation; MDA: malondialdehyde; NF-κB: nuclear factor kappa B; p.o: per os; PCNA: proliferating cell nuclear antigen; PON1: paraoxonase 1; ROS: reactive oxygen species; SLAP: Spot14-like androgen-inducible protein; SOD: superoxide dismutase; STZ: streptozotocin; TAC: total antioxidant capacity; TNF: tumor necrosis factor.

### 4.5. Effects of Royal Jelly on Male Reproductive Parameters

Egyptian bee honey (100 g) mixed with 3 g of royal jelly and 1 teaspoon of bee bread intravaginally in humans shows an increase in pregnancy rate due to increase in sperm capacitation through its antioxidant and scavenging activities against free oxygen species [104]. The administration of Egyptian royal jelly (1 g/kg bw) for 1 month increased the testicular weight and the body of epididymus, sperm count, testosterone hormone, and glutathione level, and it also caused a decrease in sperm deformity percentage, while there were no significant differences in the prostate weight, seminal vesicles, percentage of live sperm, malondialdehyde level, and body weight through the central effect of royal jelly because it contains acetylcholine [105] in hydrogen peroxide (0.5%) in drinking water induced rats. Meanwhile, 100 mg/kg of royal jelly causes a decrease in the toxic effect of cyclosporine in testis of rats due to its antioxidant property [106]. Egyptian royal jelly administered at 200, 400, or 800 mg/kg body weight once a week (6 weeks) significantly boosts testosterone level, ejaculated volume, and seminal plasma fructose; improves sperm motility and sperm total output; reduces abnormal sperm and dead sperm due to the presence of vitamin C and amino acids; and increases spermatic concentration [107].

Turkish royal jelly (50 and 100 mg/kg) for 10 days decreases the malondialdehyde level and increases superoxide dismutase, catalase, and glutathione–peroxidase activities and increases the weights of testes, epididymis, seminal vesicles, and prostate along with epididymal sperm concentration and motility in cisplatin-induced in rats. Similarly, 50, 100, or 150 mg of Chinese royal jelly/kg twice per week, respectively, administered over a 20-week period shows a significant increase (*p* < 0.05) in rabbits’ sperm concentration, total sperm output, sperm motility, live sperm, and normal sperm in rabbits; it was suggested that amino acids and vitamins might have played a role [108]. Egyptian royal jelly (0.4%) and heparin administered to buffalo induces sperm acrosome reaction but also is effective for the in vitro fertilizing capacity of the cryopreserved buffalo spermatozoa as a result of possessing motility stimulants such as adenosine and adenosine monophosphate [7].

Iranian royal jelly (100 mg/kg bw) increases testicular weight, sperm count, motility, viability, and serum testosterone levels and decreases observed sperm deformity, DNA integrity, chromatin quality, and tissue MDA levels in streptozotocin-induced diabetic rats. This might be because of its antioxidant properties due to the presence of vitamins E and C [109]. Similarly, bleomycin-induced rats treated with Iranian royal jelly (100 mg/kg/day) for 48 days improved sperm parameters and testosterone levels as well as decreased MDA levels due to its antioxidant properties [110]. On the other hand, Iranian royal jelly of 0, 50, 100, and 150 mg/kg bw increases sperm and causes a significant upregulation of transcription factor E2f1 mRNA in taxol-induced toxicity [111]. Japanese royal jelly of 50 μg/g diet or 500 μg/g diet for 12 weeks increases the intensity of spermatogenesis and testosterone levels in hamsters via its antioxidant activity [112]. Japanese royal jelly (300 mg) administered for 6 months accelerates the conversion of DHEA-S to testosterone [113], while Turkish royal jelly of 400 mg/kg daily for 4 weeks caused caspase-3-positive cells to be significantly decreased in testicular apoptosis via its anti-apoptotic activity [114]. Twenty-eight adult Wistar rats administered with royal jelly (100 mg/kg bw) for 6 weeks showed increases in CAT and FRAP activities [115]. Rats induced with hydroxylurea (225 or 450 mg kg/bw/day) followed by administration of royal jelly (100 mg kg/bw/day) for 60 days revealed improved sperm quality, hormonal, and antioxidant status as well as histology architecture [116] (Table 5).

**Table 5:** Effects of royal jelly on male reproductive parameters.

s/*n*	Bee Products	Dose/Duration of Treatment	Substance Used to Induce Stress	Animal Model Used	Route of Administration	Standard Drug	Effect on Reproductive Function Parameters	Possible Molecular Mechanisms	References
1.	Royal jelly (Iraq)	1 g/kg bw (1 month)	hydrogen peroxide (0.5%) in drinking water		Oral	-	↑ Testicular weight and the body of epididymis, sperm count, testosterone hormone and glutathione levels; ↓ sperm deformity percentage, while there were no significant differences in the prostate weight, seminal vesicles, the percentage of live sperm, MDA level, and body weight	Central effect of royal jelly because it contains acetylcholine	[105]
2.	Royal jelly (Iraq)	100 mg/kg (5, 10, and 15 days	20, 40, and 60 m/kg cyclosporine A for 5, 10 and 15 days (i.p)	Rats	Oral	-	↓ Toxic effect	Antitumor, antioxidant	[106]
3.	Royal jelly (Egypt)	200, 400, or 800 mg royal jelly (RJ)/kg body weight once a week (6 weeks)	-	Rabbits	Oral	-	↑ Testosterone level, ejaculated volume, seminal plasma fructose, improves sperm motility, sperm total output, ↓ abnormal sperm, and dead sperm	Presence of vitamin C and amino acids have increased spermatic concentration	[107]
4.	Royal jelly (Turkey)	50 and 100 mg/kg (10 days)	Cisplatin (single dose of 7 mg/kg i.p)	Rats	Oral	-	↓ MDA level and ↑ SOD, catalase, and glutathione peroxidase activities and weights of testes, epididymides, seminal vesicles, and prostate along with epididymal sperm concentration and motility	Antioxidant property	[117]
5.	Royal jelly (Japan)	50 μg/g diet or 500 μg/g diet for 12 weeks	-	Hamsters	Oral (food)	-	↑ Intensity of spermatogenesis and testosterone levels	Inhibited the age-associated decline and testosterone-secreting cells	[112]
6.	Royal jelly (Turkey)	(400 mg/kg daily for 4 weeks)	a single intraperitoneal injection of STZ (60 mg/kg)	Rats	Oral	-	↓ Caspase-3-positive cells in testicular apoptosis	Estrogenic effect	[114]
7.	Royal jelly (Chinese)	50, 100, or 150 mg of Chinese royal jelly (RJ)/kg twice per week, respectively, over a 20-week period	temperatures ranging from 23 to 36 °C	Rabbits	Oral	-	↑ Sperm concentration, total sperm output, sperm motility, live sperm, and normal sperm	Amino acids and vitamins may play a role	[108]
8.	Royal jelly (Egypt)	100 g of Egyptian bee honey mixed with 3 g of royal jelly and 1 teaspoon of bee bread	Asthenozoospermia	Humans	Intravaginal	-	↑ Pregnancy rate due to ↑ in sperm capacitation	Antioxidant and scavenging activities against free oxygen species	[104]
9.	Royal jelly (Egypt)	0.4% royal jelly + heparin	-	Buffalo (Bubalus Bubalis)	IVF	-	Induces sperm acrosome reaction but also is effective for in vitro fertilizing capacity of the cryopreserved buffalo spermatozoa	Contain motility stimulants such as adenosine and adenosine monophosphate ((AMP) N (1)-oxide)	[7]
10.	Royal jelly (Iran)	100 mg/kg bw	Streptozotocin (STZ) 60 mg/kg body weight (BW) i.p	Rats	Oral	-	↑ Testicular weight, sperm count, motility, viability, and serum testosterone levels and ↑ sperm deformity, DNA integrity, chromatin quality, and tissue MDA levels	Antioxidant activity due to the presence of vitamins E and C	[109]
11.	Royal jelly (Japan)	300 mg (6 months)	-	Human voluntiers	Oral	-	Accelerates conversion from DHEA-S to testosterone	Antioxidant activity	[113]
13.	Royal jelly (Iran)	100 mg/kg daily (48 days)	Bleomycin group (BLG) received BL (10 mg/kg twice a week) with i.p for 48 days	Rats	Oral	-	Improves bleomycin-induced toxicity on sperm parameters, testosterone, and MDA concentrations	Antioxidant activity	[110]
14.	Royal jelly (Iran)	(0, 50, 100, and 150 mg/kg bw)	Taxol 7.5 mg/kg body weight (bw), weekly	Rats	Oral	-	↑ Sperm and significant upregulation of transcription factor E2f1 mRNA	Antioxidant activity	[111]

ADP: adenosine diphosphate; AlCl_3_: aluminum chloride; bw: body weight; CAT: catalase; CCL: carbon tetra chloride; DHEA-S: dehydroepiandrosterone sulfate; DNA: deoxyribonucleic acid; FeSO_4_: ferrous sulfate; FSH: follicle-stimulating hormone; GPx: glutathione peroxidase; GR: glutathione reductase; GSH: glutathione; GST: glutathione-S-transferase; HSP 70: heat shock protein 70; i.p: intraperitoneal; IL: interleukin; IVF: in vitro fertilization; LDH: lactate dehydrogenase; LH: leutinizing hormone; L-NAME: N^ω^-nitro-l-arginine methyl ester; LPO: lipid peroxidation; MDA: malondialdehyde; NF-κB: nuclear factor kappa B; p.o: per os; PCNA: proliferating cell nuclear antigen; PON1: paraoxonase 1; ROS: reactive oxygen species; SLAP: Spot14-like androgen-inducible protein; SOD: superoxide dismutase; STZ: streptozotocin; TAC: total antioxidant capacity; TNF: tumor necrosis factor.

### 4.6. Effects of Bee Bread on Male Reproductive Parameters

The administration of 0.5 g/kg/bw Malaysian bee bread for 12 weeks caused increases in testicular antioxidant enzymes, downregulated inflammation and apoptosis, and increased PCNA immunoexpression, as well as improved lactate transport, through its antioxidant, anti-inflammatory, and antiapoptotic properties [118,119] (Table 6).

**Table 6:** Effects of bee bread on male reproductive parameters.

s/*n*	Bee Products	Dose/Duration of Treatment	Substance Used to Induce Stress	Animal Model Used	Route of Administration	Standard Drug	Effect on Reproductive Function Parameters	Possible Molecular Mechanisms	References
1.	Bee bread (Malaysia)	0.5 g/kg/day bw (12 weeks)	High-fat diet	Rats	Oral	Orlistat	Upregulated testicular antioxidant enzymes, downregulated inflammation and apoptosis, and increased PCNA immunoexpression, as well as improving lactate transport	Antioxidant, anti-inflammatory, and antiapoptotic properties	[118,119]

bw: body weight; PCNA: Proliferating cell nuclear antigen.

## 5. Conclusions and Future Directions

**Figure 2:**
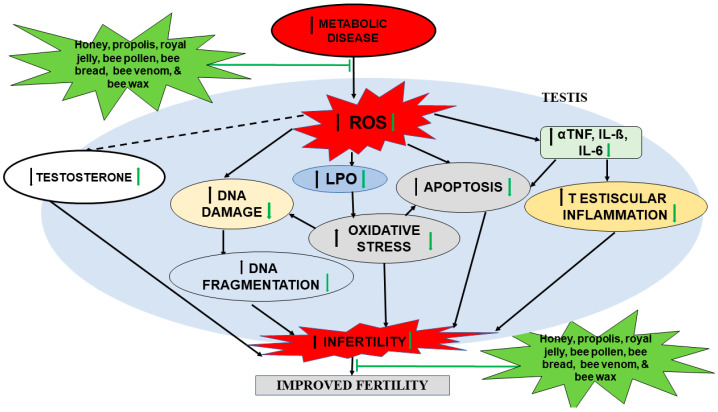
Summary of the effects of bee products on male reproductive impairment (adapted from [60]). DNA: deoxyribonucleic acid; IL-6: interleukin 6; IL-ß: interleukin ß; LPO: lipid peroxidation; αTNF: α-tumor necrotic factor; ROS: reactive oxygen species.

Bee, bee products, and apitherapy came from ancient times, but lately, apitherapy has received great attention from researchers worldwide, who have investigated their potential beneficial effects on male reproductive functions. There are numerous significant ameliorative effects for several male reproductive impairments with the treatment of varies bee products, especially in animal studies, but in general, these treatments have not been proven to be effective and safe in clinical experiments. The various extracts of bee products have shown functional biological properties due to their high content of flavonoids, polyphenols, and radical scavenging capacity, as summarized in Figure 2. However, more research including experimental and clinical studies are required to verify the effectiveness of these extracts and their underlying molecular mechanisms of actions. The main goal of apitherapy in the next few years will be to further our understanding of the developmental, scientific basis and clinical apitherapy to make it scientifically accepted for the treatment of male reproductive impairment.

## Data Availability

Not applicable.
